# Dissociating Statistically Determined Normal Cognitive Abilities and Mild Cognitive Impairment Subtypes with DCTclock

**DOI:** 10.1017/S1355617722000091

**Published:** 2022-02-21

**Authors:** Emily F. Matusz, Catherine C. Price, Melissa Lamar, Rod Swenson, Rhoda Au, Sheina Emrani, Victor Wasserman, David J. Libon, Louisa I. Thompson

**Affiliations:** 1 Department of Geriatrics and Gerontology, New Jersey Institute for Successful Aging, School of Osteopathic Medicine, Rowan University, Stratford, NJ, USA; 2 Clinical and Health Psychology, University of Florida, Gainesville, FL, USA; 3 Department of Behavioral Sciences and the Rush Alzheimer’s Disease Center, Rush University Medical Center, Chicago, IL, USA; 4 University of North Dakota School of Medicine and Health Sciences, Grand Forks, ND, USA; 5 Boston University Schools of Medicine & Public Health, Boston, MA, USA; 6 Department of Psychology, Rowan University, Stratford, NJ, USA; 7 Department of Psychiatry and Human Behavior, Alpert Medical School of Brown University, Providence, RI, USA; 8 Butler Hospital Memory & Aging Program, Providence, RI, USA

**Keywords:** Cognition, Clock drawing, Digital technologies, Digital clock drawing test, Aging, Boston process approach, Executive function

## Abstract

**Objective:**

To determine whether the DCTclock can detect differences across groups of patients seen in the memory clinic for suspected dementia.

**Method:**

Patients (*n* = 123) were classified into the following groups: cognitively normal (CN), subtle cognitive impairment (SbCI), amnestic cognitive impairment (aMCI), and mixed/dysexecutive cognitive impairment (mx/dysMCI). Nine outcome variables included a combined command/copy total score and four command and four copy indices measuring drawing efficiency, simple/complex motor operations, information processing speed, and spatial reasoning.

**Results:**

Total combined command/copy score distinguished between groups in all comparisons with medium to large effects. The mx/dysMCI group had the lowest total combined command/copy scores out of all groups. The mx/dysMCI group scored lower than the CN group on all command indices (*p* < .050, all analyses); and lower than the SbCI group on drawing efficiency (*p* = .011). The aMCI group scored lower than the CN group on spatial reasoning (*p* = .019). Smaller effect sizes were obtained for the four copy indices.

**Conclusions:**

These results suggest that DCTclock command/copy parameters can dissociate CN, SbCI, and MCI subtypes. The larger effect sizes for command clock indices suggest these metrics are sensitive in detecting early cognitive decline. Additional research with a larger sample is warranted.

## INTRODUCTION

The clock drawing test (CDT) is one of the oldest and most widely used neuropsychological tests due to its ease of administration, brevity, and ability to capture a wide range of neuropsychological functions (Cosentino et al., 2004; Libon et al., 1996). The CDT is comprised of two conditions, producing a drawing to command, followed by copying a model of a clock. Successful performance requires accessing the semantic attributes associated with a clock, the necessary linguistic abilities to translate the command for time setting into the correct graphomotor response, motor operations, spatial reasoning and organization, working memory, and the capacity for mental planning (Cosentino et al., 2004; Libon et al., 1996).

The literature using traditional paper and pencil CDT is substantial and includes many investigations of its accuracy in detecting cognitive changes in aging and neurodegenerative disease (Hazan et.al, 2018). CDT performance has also been shown to distinguish between dementia subtypes and between mild cognitive impairment (MCI) subtypes (Ahmed et al., 2016; Cosentino et al., 2004; Kozora & Cullum, 1994; Libon et al., 1996; Price et al., 2011; Royall et al., 1998). For example, Ahmed et al. (2016) found that CDT errors across MCI subtypes are highly associated with language skills, including naming and verbal concept formation. Prior research using analog scoring methods has shown that while participants with AD typically improve from the command to the copy test condition, participants with disproportionate dysexecutive impairment, as seen in vascular dementia (VaD) and Parkinson’s disease (PD) often fail to improve. This behavior is thought to be secondary to the impaired frontal systems operations that can typify these disorders (Cosentino et al., 2004; Libon et al., 1996; Price et al., 2011). Indeed, it has been shown that participants with VaD make more errors overall and tend to make the same errors on both command and copy conditions, suggesting an inability to alter mental set as test conditions change (Cosentino et al., 2004; Libon et al., 1996; Price et al., 2011). Analog clock drawing behavior has also been shown to be associated with neuroimaging biomarkers of disease. For example, Shoyama et al. (2011) obtained analog clock drawings to command from young normal controls and assessed brain activity using multichannel near-infrared spectroscopy. These investigators found that total time to completion was correlated with increased prefrontal oxygen hemoglobin recruitment.

Despite its merits and longevity, the traditional CDT pose some challenges as a diagnostic and screening tool. For example, standard 3, 5, or 10-point scoring systems tend to capture only a small number of features or errors that might indicate cognitive impairment. Additional problems associated with analog clock drawing scoring systems revolve around the need to establish inter-rater reliability and the time necessary to score protocols (Price et al., 2011). These problems tend to limit how the CDT could be used in settings such as primary medical care to screen for neurocognitive impairment.

Over the past decade, innovations in digital technology have enabled researchers to create a digital clock drawing test (dCDT) that captures a wide range of clock drawing behavior in real time yielding thousands of variables or features (Libon et al., 2014; Müller et al.,2019; Schejter-Margalit et al., 2021; Souillard-Mandar et al., 2016). Recent research has shown that machine learning algorithms using features extracted from the dCDT are able to classify dementia and non-dementia patients into their respective groups (Binaco et al., 2020; Souillard-Mandar et al., 2016; Dion et al., 2020). For example, Bianco et al. (2020) analyzed digital clock drawing features using machine learning algorithms. In this research, neural networks employing an information theoretic feature selection approach was able to achieve the best 2-group classification at or above 83% between patients diagnosed with AD versus and MCI; and between amnestic versus mixed/ dysexecutive MCI, and between CN versus amnestic or mixed/dysexecutive MCI subtypes. In another study, Davoudi et al. (2021) extracted digital clock drawing kinematic, time-based, and visuospatial features and examined how well these features could classify AD, VaD, and normal control participants into their respective groups. Optimal area under the curve was achieved using a combination of command and copy variables measuring kinematic (mean pen pressure, ratio of pen pressure to velocity), time-based, and graphomotor features.

Perhaps some of the most innovative and potentially informative data that can be extracted using the dCDT are the variety of timed-based parameters. For example, prior research has revealed that the majority of clock drawing time is spent not actually drawing. This behavior–called *think time* or time spent not putting ink on the test form has been demonstrated in patients with multiple sclerosis (Libon et al., 2014), MCI (Dion et al., 2020), and community volunteers evaluated as part of the Framingham Heart Study (Piers et al., 2017). Digital clock drawing research has also uncovered a number of decision-making latency variables defined by the time elapsed between clock drawing components (Libon et al., 2014, Piers et al., 2017). Research shows that these *decision-making* latencies vary in the command versus the copy test condition (Libon et al., 2014; Piers et al., 2017). Another recent validation study in non-demented older adults found that total clock drawing time *positively* correlated with performance in multiple cognitive domains, while selected decision-making latencies were *negatively* correlated with performance on many of the same tasks (Dion et al., 2020).

Behavior often seen on the CDT includes the tendency of patients to initiate the drawing of numbers inside the clock face using anchor digits (i.e., the numbers 12, 6, 3, 9). Lamar et al. (2016) studied cognitively normal (CN) older adults who use an anchoring organizational strategy involving key digits of the clock face (numbers 12, 3, 6, and 9). Participants using this strategy had better performance on executive and memory tasks and exhibited greater regional integration within the left orbitofrontal and temporal cortices and the right anterior cingulate/right frontal gyrus.

In sum, there is growing support that digital clock drawing metrics aid in the differential diagnosis of cognitive diseases of aging and underlying disruptions in brain function. However, the dCDT (Libon et al., 2014; Souillard-Mandar et al., 2016) require some post-processing. Moreover, normative data is limited. To help make the transition from research to widespread clinical use it would be useful for a test to require little to no post-processing and clock drawing indices expressedas standard scores measuring constructsthat underlie successful performance. Recently, a dCDT, DCTclock^™^, has become commercially available as part of the Linus health platform (https://linus.health). The DCTclock^™^ builds upon previous dCDT research, capturing metrics previously described in the literature (e.g., ‘*think time*,’ spatial organization, drawing size) with machine learning analytics. The DCTclock^™^ diverges from prior dCDT by introducing a cloud-based scoring platform requiring no examiner post-processing, four age-adjusted composite scores for both command and copy conditions, and a composite total command/copy score designed to be user friendly and aid clinical interpretation. In a recent paper, Rentz and colleagues (2021) studied a group of CN participants who had amyloid and tau positron emission tomography (PET) imaging and a group of participants with MCI or early AD. Among participants with imaging biomarkers of amyloid and tau, the DCTclock^™^ total score and spatial reasoning index scores were associated with greater amyloid and tau burden. Despite these interesting findings, there is limited research on these DCTclock^™^ metrics to date.

The current study sought to further investigate the utility of DCTclock^™^ generated metrics for distinguishing between statistically defined MCI subtypes. In the current study, the DCTclock^™^ was administered to memory clinic patients who were classified as presenting with subtle cognitive impairment (SbCI; Edmonds et al., 2015) or MCI using statistically determined criteria (Bondi et al., 2014; Jak-Bondi et al., 2009). Past research regarding MCI suggests different clinical and pathology outcomes depending on specific MCI subtypes (Schneider et al., 2009). Therefore, a test that is able to dissociate between MCI subtypes would have considerable utility in both primary and special care settings. The goal of the current research was to assess differences across participant groups in the total score and composite metrics generated by DCTclock^™^.

## METHODS

### Participants

Participants in the current research (*n* = 103; 100% White older adults) were patients recruited from Rowan University, New Jersey Institute for Successful Aging, Memory Assessment Program (MAP). All MAP patients underwent a comprehensive neuropsychological evaluation and were examined by a social worker and board-certified geriatric psychiatrist. An Magnetic resonance imaging (MRI) study of the brain and appropriate serum blood tests were obtained to evaluate for reversible causes of dementia. A clinical diagnosis was determined for each patient at an interdisciplinary team conference. Participants diagnosed with MCI presented with subjective cognitive complaints and/or evidence of cognitive impairment relative to age and education, preservation of general functional abilities, and the absence of dementia. Participants were excluded if there was any history of head injury, substance abuse, or major psychiatric disorders, including major depression, epilepsy, B12, folate, or thyroid deficiency. For all participants, a knowledgeable family member was available to provide information regarding functional status. This study was approved by the Rowan University Institutional Review Board with consent obtained consistent with the Declaration of Helsinki.

### Neuropsychological Assessment

The neuropsychological protocol used to classify MCI subtype assessed three domains of cognition: executive control, naming/lexical access, and episodic memory. Measures of visuospatial functioning were not assessed or used for MCI subtype classification. From this protocol, nine parameters, three from each neurocognitive domain, were used to classify MCI subtype as described below (Emrani et al., 2018). All test scores were expressed as z-scores derived from normative data.

#### Executive Control

This cognitive domain was assessed with three tests including The Boston Revision of the Wechsler Memory Scale-Mental Control subtest (Lamar et al., 2002); the letter fluency test (Spreen & Strauss, 1990); and the Trail Making Test-Part B (Reitan & Wolfson, 1985). The dependent variable for the mental control subtest was the total non-automatized accuracy index (see Lamar et al., 2002 for full details). The dependent variables obtained from the letter fluency test and Trail Making Test-Part B were demographically corrected scores provided by Heaton et al. (2004).

#### Lexical Access/Language

This domain was also assessed with three tests, including the 60-item version of the Boston Naming Test (BNT) (Kaplan et al., 1983); a test of semantic (‘animals’) fluency where participants were asked to produce as many names of animals in 60s excluding perseverations and extra-category intrusion responses (Spreen & Strauss, 1990); and the Wechsler Adult Intelligence Scale-III (WAIS-III) Similarities subtest (Wechsler, 1997). The dependent variables for the BNT and ‘animal’ fluency tests were standard scores adjusted for age, sex, and race obtained from Heaton et al. (2004). The dependent variable obtained from the WAIS-III Similarities subtest was the age-corrected scale score (Wechsler, 1997).

#### Memory and Learning

This cognitive domain was assessed with the 9-word California Verbal Learning Test (CVLT)-Mental Status test (Delis et al., 2000). This test was scored and administered using standard instructions. Three CVLT-short form variables were used in the current research including total immediate free recall, delayed free recall, and the delayed recognition measure adjusted for age, sex, and education.

### Determination of Clinical Subtypes

#### Single and Multi-Domain MCI.

Jak et al. (2009) criteria were used to determine MCI subtype. Single domain MCI was diagnosed when participants scored >1.0 standard deviation below normative expectations on any of two of the three measures within a single cognitive domain. Mixed MCI was diagnosed when participants scored >1.0 standard deviation below normative expectations on any of two of the three measures within two or more cognitive domains. Based on these procedures, 21 participants were diagnosed with single domain amnestic MCI (aMCI), 6 participants were diagnosed with single domain dysexecutive MCI, and 22 were diagnosed with mixed or multi-domain MCI. Because of the small number of dysexecutive MCI participants, a combined mixed/dysexecutive (mx/dys) MCI subgroup (*n* = 28) was constructed. This decision was made based on prior research (Emrani et al., 2018, Eppig et al., 2012; Libon et al., 2011) where mixed/dysexecutive participants presented with similar patterns of impairment on executive tests. Table 1 shows descriptive statistics for neuropsychological performance, demographics, and clinical ratings in each group.

#### SbCI.

Thirty-three of the 54 participants not meeting Jak et al. (2009) criteria for MCI were classified as presenting with subtle MCI (SbCI) using a modification of Edmonds et al. (2015) criteria. These participants scored >1 sd below the mean on two of the nine neuropsychological measures in different cognitive domains (Edmonds et al., 2015).

#### Cognitive Normal (CN) Group

Twenty-one participants did not meet criteria for either SbCI (Edmonds et al., 2015) or MCI (Bondi et al., 2014; Jak et al., 2009). One individual presented with some, but very little cognitive impairment, such that only one of the nine neuropsychological parameters was below the 1 SD cutoff. All of these participants were combined into a single group and labeled as presenting with CN.

### The dCDT

DCTclock^™^ is based on the traditional paper and pencil clock drawing task and was originally designed, with cooperation from the Clock Sketch Consortium (Libon et al., 2014), at Lahey Clinic and the Massachusetts Institute of Technology (Souillard-Mandar et al., 2016). It was further developed and licensed for research use by Digital Cognition Technologies Inc. now part of Linus Health, and is cleared by the Food and Drug Administration for cognitive assessment. Participants are presented with a paper test form containing a faint dot pattern and handed a digital pen that looks and functions like a normal pen but contains a camera sensor that captures pen position every 12 ms. The instructions used to administer the DCTclock^™^ are consistent with traditional CDT administration and included both command and copy test conditions. In the command condition, participants are asked to, “draw the face of a clock, put in all numbers, and set the hands for *10 after 11*.” Upon completion of the command test condition, the copy test condition is administered whereby participants are asked to copy a model of a clock with hands set for ‘*10 after 11*’.’The digital pen allows for the capture of *thousands* of clock drawing features to be analyzed as a series of timestamped (x,y) coordinates.

DCTclock^™^ produces multiple objective measurements that were derived from approximately 5000 digital clock drawings using machine learning algorithms (Binaco et al., 2020; Davis et al., 2014). Machine learning algorithms were previously developed to calculate meaningful clock scores based on their ability to discriminate performance between housands of healthy controls and participants from different diagnostic groups including aMCI, AD dementia, PD and other neurodegenerative disorders (Davis et al., 2014; Souillard-Mandar et al., 2016). Details on how the DCTclock^™^ algorithm and scoring process have been described in detail elsewhere (Rentz et al., 2021). Table 2 contains a description of the 9 DCTclock^™^ indices used for this analysis.

### Statistical Analyses

Hierarchical linear regression models with block-wise predictor entry were constructed to investigate differences among groups on the DCTclock^™^ composite indices, and the total command/copy score. Due to the lack of normative data for education and sex for the composite indices, education and sex were entered into Step 1 of the hierarchical models for these variables to allow for the interpretation of group differences after controlling for variability among these factors. Unlike the composite indices, the DCTclock^™^ total command/copy score is not adjusted for age, and therefore we entered age, in addition to education and sex, in Step 1 of the hierarchical models using the total command/copy score. In Step 2, dummy coded variables representing between-group differences among the CN and MCI group subtypes were entered into the model. Dummy coding is a method frequently utilized in regression analysis to allow for the coding and incorporation of categorical predictors into the model (see Tabachnick & Fidell, 2013 for details). This coding sets one level of the variable as the control group, to which all other groups are then compared. In order to obtain a description of all possible group differences, *K* − 1 dummy codes, where *K* represents the levels of the categorical predictor, were created and included into the regression analysis. The results produced from Step 2 were interpreted to assess for between-group differences after controlling for demographics.

## RESULTS

### Preliminary Analyses

Four participants with DCTclock^™^ composite index scores in excess of 3.29 were identified as outliers and removed from analyses (Tabachnick & Fidell, 2013). No violations associated with the ordinary least squares estimator were identified. Descriptive statistics for all nine DCTclock^™^ parameters can be found in Table 3.

### Hierarchical Regression Analyses

#### Distinguishing SbCI from CN

DCTclock^™^ measures that significantly distinguished SbCI participants from CN participants include: the Digital Cognition Technologies (DCT) total score (*t* = −2.85, *p* = .005), command spatial reasoning (*t* = −2.40, *p* = .018), copy drawing efficiency (*t* =−2.08, *p* = .04), copy information processing (*t* =−2.28, *p* = .025), and copy simple motor (*t* =−2.13, *p* = .035). For all of these measures participants in the SbCI group obtained lower scores than those in the CN group (Table 4). Overall, the model for DCT total score generated the greatest effect size (*β*=−0.33).

#### Distinguishing MCI from CN

The DCTclock^™^ measures that significantly distinguished MCI from CN participants included: the DCT total score (aMCI: *t* = −2.07, *p* = .005; mx/dysMCI: *t* =−5.07, *p* < .001), command drawing efficiency (mx/dysMCI: *t* = −3.19, *p* = .002), command information processing (aMCI: *t* = −2.23, *p* = .028; mx/dysMCI: *t* = −2.77, *p* = .007), command spatial reasoning (aMCI: *t* =−3.01, *p* = .003; mx/dysMCI: *t* =−5.04, *p* < .001), copy drawing efficiency (mx/dysMCI: *t* =−3.13, *p* = .002), and copy information processing (mx/dysMCI: *t* =−2.97, *p* = .004).

Overall, of the three indices that distinguished aMCI from CN (total score, command information processing, and command spatial reasoning), the greatest effect size was achieved using the command spatial reason index (*β* = −.35). Of the six indices that distinguished mx/dysMCI from CN (total score, command drawing efficiency, command information processing, command spatial reasoning, copy drawing efficiency, copy information processing), the greatest effect size was achieved using the DCT total score and command spatial reason index (both *β* =−.59). None of the copy indices significantly distinguished aMCI from CN participants (*p* >.05).

#### Distinguishing mx/dysMCI from aMCI.

DCTclock^™^ measures that significantly distinguished mx/dysMCI participants from aMCI participants included DCT total score (*t* =−2.82, *p* = .006), copy drawing efficiency (*t* =−2.11, *p* = .037), and copy Spatial Reasoning (*t* = −2.54, *p* = .012). Participants in the mx/dysMCI group scored lower on these three indices compared to those in the aMCI group. Overall, DCT total score and the copy spatial reasoning index score generated the greatest effect size (both *β*s =−0.33).

#### Distinguishing SbCI from MCI and CN

DCTclock^™^ measures that significantly distinguished SbCI participants from CN participants included the DCT total score (*t* = 2.85, *p* = .005), the command condition spatial reasoning index (*t* = 2.41, *p* = .018), and the copy condition drawing efficiency (*t* = 2.09, *p* = .04) and simple motor indices (*t* = 2.14, *p* = .035). Only the command condition spatial reasoning index distinguished SbCI from mx/dsyMCI (*t* = 3.04, *p* = .003) and none of the scores distinguished ScCI from aMCI.

## DISCUSSION

Our findings suggest that DCTclock^™^ metrics can accurately distinguish between Jak/Bondi neuropsychological-defined clinical MCI subtypes, SbCI , and normal cognitive aging in a memory clinic sample. Critically, while individual index scores varied from one group comparison to the next, the DCTclock^™^ Total Score, a single score that aggregates across all command and copy condition metrics revealed significant differences in performance patterns across groups.

In previous research Cosentino et al. (2004) found that clock drawing errors in the command condition were associated with overall illness severity and degrade access to semantic knowledge. Errors produced in the copy test condition were associated with dysexecutive difficulty. These findings underscore the complimentary, but different neurocognitive abilities that underlie successful clock drawing. It is very likely that these neurocognitive disabilities contribute to a reduced DCTclock^™^ total ccore. More research is necessary to test this supposition. Nonetheless, the current research suggests that the DCTclock^™^ total score could be a reasonable omnibus measure to screen for many of the important cognitive domains that underlie MCI and SbCI.

It has been suggested that the biological substrate underlying insidious onset AD/VaD spectrum syndromes (Emrani et al., 2021a) have their origin years before clinical symptoms emerge. Thus, there is an urgent need to develop effective and time efficient tests to screen for emergent neurodegenerative illness. The traditional venue to assess for pre-dementia/ dementia illness has been the specialty memory clinic. Yet, the worldwide prevalence of dementia syndromes, such as AD, suggests that screening for dementia should become part of *routine primary care*. The brevity, ease of administration, autonomously scoring, and sensitivity to AD biomarkers suggests DCTclock^™^ could provide the means to screen for neurocognitive impairment in the primary care environment.

In addition to the total score, we found evidence that DCTclock^™^ index scores that capture more nuanced aspects of clock drawing performance also have utility, particularly for distinguishing between cognitive profiles to inform differential diagnosis. The index score with the best dissociation between CN versus SbCI and Jak/Bondi determined MCI groups in our sample was command spatial reasoning index, a compilation measuring clock face circularity and the spatial relationships of the components drawn within the clock face (i.e., digits, clock hands). These data suggest that nuanced changes in motor, executive, and visuospatial functioning critical for clock organization and construction may characterize SbCI and distinguish between profiles of early-stage amnestic versus executive cognitive decline. These findings lend empirical support to prior research showing heteromodal ventral stream alterations in normal control participants who did not use anchor digits to organize their clock drawings, thereby displaying less spatial organization/reasoning in their approach (Lamar et al., 2016). If looked at longitudinally, subtle motor and spatial reasoning deficits may be a harbinger of cognitive impairment given the role of ventral steam visual processing regions in signaling the emergence of SbCI and conversion from normal cognition to MCI and then to AD (Lee et al., 2008; Thomann et al., 2008). The findings of the current study also add to data reported by Rentz and colleagues (2021), who showed that the spatial reasoning index score was associated with greater cerebral amyloid and tau burden. Interestingly, however, their finding was also specific to the spatial reasoning index score but from the copy condition rather than the command condition.

In the current study, copy condition index scores tended to have the most utility when distinguishing SbCIfrom normal cognition, and distinguishing mx/dysexecutive MCI from amnestic MCI and normal cognition. Of the various index scores in the copy condition, comparisons of group performance, with the exception of the amnestic MCI versus normal cognition comparison, most consistently differed on the drawing efficiency index. Consistent with the findings reported by Cosentino et al. (2004), these findings might suggest that clock drawing to copy is specifically linked to dysexecutive difficulty. Group differences on copy condition index scores underscores the benefit of this test condition and is consistent with a large corpus of prior clock drawing literature (see Cosentino et al., 2004; Price et al., 2011; Wiggins et al., 2021). However, further research and replication will be needed to parse the differential contributions of the command versus copy conditions for DCTclock^™^ indices in normal aging and early-stages cognitive decline. Further research is also needed to clarify the potentially different ways in which DCTclock^™^ index scores relate to incident clinical cognitive profiles and neurodegenerative disease biomarkers.

The current research adds to a growing body of research demonstrating how digital technology can be engineered to extract and define subtle and very nuanced behavior that can differentiate between pre-MCI and MCI subtypes (Emrani et al., 2021a, 2021b) resulting in better diagnostic decision-making. Previous research has suggested that participants diagnosed with amnestic MCI may be at greater risk to progress to pathological confirmed AD (Guillozet et al., 2003; Grundmanet al., 2004; Devlin et al., 2021). Patients with mixed or dysexecutive MCI may be expected to revert to a CN state or progress to other dementia syndromes such as frontotemporal dementia, dementia with Lewy bodies, VaD associated with small vessel disease, or depression (Schneider et al., 2009; Ferman et al., 2013; Dugger et al., 2015).

The current research is not without limitations. First, our sample size is modest, overwhelming white, and highly educated, which limits the generalizability of our findings. Gathering digital clock protocols from ethnically and racially diverse patients and non-native English speakers is critical. Also, the exact relation between DCTclock^™^ indices and education needs to be determined. Second, data were collected from self-referrals presenting to a specialized memory and aging program because of memory concerns. As stated above, to maximize the effectiveness for any neurocognitive screening test, data need to be collected in diverse setting such as primary medical care, family medicine, and obstetrics/gynecology where many women get their primary care. Third, biomarkers such as cerebral or cerebral spinal fluid (CSF) amyloid and tau levels, or brain volumetrics and vascular disease markers were not available for this analysis. We therefore cannot confirm the distinctness of our Jak/Bondi defined clinical groups, and how these groups relate to neurodegenerative neuropathology or cerebrovascular disease. In this regard, there is a need to gather DCTclock^™^ data on diverse clinical samples with dementia biomarkers for further validation. Fourth, we acknowledge that other neuropsychological tests/domains of cognitive functioning could have been used for classification of MCI groups. The rationale for using the protocol that we did was based on prior research showing that the specific neuropsychological tests used were able to illustrate key neurocognitive constructs and differentiate between MCI subtypes (Emrani et al., 2018). Moreover, in addition to Jak-Bondi criteria others mean to classify MCI patients in relation to DCT^™^ performance should be undertaken. Lastly, the current study lacks the inclusion of test data from the visuospatial functioning domain. This domain is relevant to clock drawing performance, and should be examined in the future.

Despite these limitations, the current study contributes to the literature in that this is the first report on the ability of DCTclock^™^ metrics to distinguish between CN and clinical SbCI/MCI subtypes. The data described above, along with recent findings described by Rentz and colleagues (2021) build upon years of prior digital clock drawing and machine learning research (Binaco et al., 2020; Lamar et al., 2016; Libon et al., 2014; Piers et al., 2017; Souillard-Mandar et al., 2016). Collectively, these data provide evidence for a commercialized and Federal drug administration (FDA)-approved digital clock drawing tool, DCTclock^™^ that has the capacity to leverage this technology for broader clinical and research use. The provision of normative data, automated scoring, and simplified composite metrics from the DCTclock^™^ system may improve the usability and efficiency of machine learning-based analytics of clock drawing performance. Finally, a tablet-based version of the DCTclock has recently been developed by Linus Health. As the field moves further toward tablet-based digital assessment, additional research is needed to investigate and compare the validity of digital pen versus table-based approaches to clock drawing assessment.

## Supplementary Material

supplemental tables

## Figures and Tables

**Table 1.** Characteristics of neuropsychologically-defined clinical MCI subgroups

*a. Neuropsychological test performance: means z-scores and standard deviations*				

Tests by cognitive domain	CN	SbCI	aMCI	mx/dysMCI

*Executive control*				
WMS-Mental Control	.15(.58)	−.20(.64)	−.06(.72)	−.95(1.14)
Letter fluency (‘FAS’)	.35(1.03)	−.45(1.06)	−.20(64)	−1.45(1.01)
Trail Making Test – Part B	.20(.77)	−.29(.67)	−.19(1.00)	−.91(.82)
*Lexical access/language*				
Boston Naming Test (BNT)	.47(.93)	−.01(.97)	.15(.77)	−1.12(1.31)
Semantic fluency (animals)	−.08(.65)	−.78(92)	−1.17(.93)	−1.87(.97)
WAIS-III – Similarities	.56(.79)	.41(.76)	.00(.92)	−.48(.84)
*Memory & learning*				
CVLT-Short form immediate free recall	.50(.95)	−.10(.79)	−.77(.82)	−.95 (.80)
CVLT-Short form delayed free recall	.60(.74)	−1.14(1.12)	−1.74(.49)	−1.09(1.20)
CVLT-Short form delayed recognition	.60(1.18)	−.06(.87)	−1.52(.73)	−1.15(1.08)

*b. Demographic and clinical information: means and standard deviations*				

Demographic and clinical variables	CN	SbCI	aMCI	mx/dysMCI

Age	76.1(5.82)	75.9(7.73)	73.9(6.67)	75.4(6.04)
Education	15.2(2.93)	15.4(2.18)	13.8(2.16)	14.4(2.36)
MMSE	28.5(1.50)	27.9(1.75)	26.7(2.35)	26.7(1.70)
WRAT-IV reading subtest	117.1(15.48)	113.6(15.83)	112.1(14.20)	107.3(15.29)
Geriatric Depression Scale	3.0 (3.07)	3.5(2.71)	2.9(2.24)	4.1(2.49)

Note: WMS = Wechsler Memory Scale; WAIS-III = Wechsler Adult Intelligence Test; CVLT = California Verbal Learning Test; MMSE = Mini-Mental State Examination; WRAT-IV = Wide Range Achievement Test. CN = normal cognition; SbCI = subtle cognitive impairment; aMCI = amnestic mild cognitive impairment; mx/dysMCI = mixed/dysexecutive mild cognitive impairment.

**Table 2.** Neurocognitive Biomarkers captured with the DCTclock

Total Score	A single score between 0 and 100 that captures overall performance across command and copy conditions
Composite Scales Drawing Efficiency	The efficiency the participant demonstrated during the process of drawing the clock. This considers metrics such as total time spent compared to amount of ink used, pen strokes and ink length, size of the drawing, etc.
Simple/Complex Motor Operations	The motor components involved during the process of drawing the clock. This considers metrics including speed and oscillatory motion and can be helpful in parsing out graphomotor concerns.
Information Processing Speed	The ability to process information demonstrated during the process of drawing the clock. This considers metrics including latencies, pauses, and relative time spent thinking (without pen to paper) versus actively drawing.
Spatial Reasoning	The spatial abilities demonstrated during the process of drawing the clock. This considers metrics including geometric and spatial placement of the various properties of the drawing.

Note: Score calculation is automated and cloud-based. Composite and subscale scores are calculated for both command and copy conditions and normed with respect to cognitively healthy individuals. Composite scales and subscale metrics are adjusted for age.

**Table 3.** DCTclock indices (descriptive statistics)

	CN, *Mean (SD)*	SbCI, *Mean (SD)*	aMCI, *Mean (SD)*	mx/dys MCI, *Mean (SD)*

Total Command/Copy Score (*t*-score)	65.35 (19.50)	47.01 (20.41)	59.91 (24.45)	40.72 (20.93)
**Command Indices (*z*-score)**				
Drawing Efficiency	−0.08 (1.37)	−1.22 (1.17)	−0.75 (1.35)	−1.39 (1.68)
Information Process Speed	0.40 (0.97)	−0.33 (1.27)	−0.23 (1.03)	−0.34 (0.99)
Simple/Complex Motor	−0.69 (1.12)	−1.29 (0.94)	−0.77 (1.07)	−1.13 (1.26)
Spatial Reasoning	−0.48 (1.10)	−1.30 (1.29)	−1.39 (1.35)	−1.21 (1.39)
**Copy Indices (*z*-score**)				
Drawing Efficiency	0.18 (0.94)	−0.96 (1.31)	−0.33 (1.11)	−1.01 (1.34)
Information Process Speed	−0.22 (−0.90)	−0.58 (1.18)	−0.02 (0.86)	−0.58 (0.97)
Simple/Complex Motor	−0.28 (1.09)	−0.70 (0.99)	−0.28 (0.90)	−0.60 (1.03)
Spatial Reasoning	−0.96 (1.33)	−1.13 (0.86)	−0.63 (1.15)	−1.55 (1.30)

*Note.* CN = cognitively normal; SbCI = subtle cognitive impairment; aMCI = amnestic cognitive impairment; mx/dysMCI = mild/dysexecutive cognitive impairment.

**Table 4.** DCTclock hierarchical regression analysis summary

Outcome	Step	Predictor Variable	*R* ^2^	Δ*R*^2^	*B*	*SE* (*B*)	95% CI (*B*)	*β*	*sr* ^2^

DCTclock Total Score	1	Age	.05	.05	−.79	.35	[−1.48, .10]	−.23	.05
		Education			.18	.95	[−1.71, 2.07]	.02	.00
		Sex^a^			.37	3.75	[−7.07, 7.82]	.01	.00
	2		.26***	.205***					
		SbCI/CN			−16.50	5.79	[−27.99, −5.01]	−.33**	.06
		aMCI/CN			−13.67	6.58	[−26.72, −.62]	−.24*	.03
		mx-dysMCI/CN			−30.63	6.03	[−42.60, −18.66]	−.59***	.20
		aMCI/SbCI			2.83	6.00	[−9.01, 14.74]	.05	.00
		mx-dysMCI/SbCI			−14.13	5.41	[−24.86, −3.39]	−.27*	.05
		mx-dysMCI/aMCI			−16.96	6.01	[−28.89, −5.02]	−.33**	.06
COM-DE	1		.08*	.08*					
		Education			.17	.06	[.05, .29]	.27**	.07
		Sex^a^			.21	.24	[−.27, .69]	.04	.01
	2		.17**	.09*					
		SbCI/CN			−.75	0.40	[−1.54, .05]	−.23	.03
		aMCI/CN			−.58	.45	[−1.47, .31]	−.16	.01
		mxMCI/CN			−1.32	.42	[−2.15, .50]	−.39**	.09
		aMCI/SbCI			.16	.41	[−.65, .98]	.04	.00
		mxMCI/SbCI			−.58	.37	[−1.32, .16]	−.17	.02
		mxMCI/aMCI			−.74	.41	[.08, −1.56]	−.22	.03
COM-IP	1		.00	.00					
		Education			.03	.04	[−.06, .12]	.07	.00
		sex^a^			−.09	.18	[−.44, .26]	−.05	.00
	2		.09	.08*					
		SbCI/CN			−.54	.30	[−1.13, .05]	−.24	.03
		aMCI/CN			−.75	.33	[−1.41, −.08]	−.28*	.05
		mxMCI/CN			−.86	.31	[−1.47, −.24]	−.35**	.07
		aMCI/SbCI			−.20	.30	[−.81, .40]	−.08	.00
		mxMCI/SbCI			−.31	.28	[−.86, .24]	−.13	.01
		mxMCI/aMCI			−.11	.31	[−.72, .50]	−.05	.00
COM-SCM	1		.03	.03					
		Education			.02	.05	[−.07, .11]	.05	.00
		Sex^a^			.30	.19	[−.07, .66]	.16	.03
	2		.08	.05					
		SbCI/CN			−.60	.32	[−1.23, .02]	−.25	.04
		aMCI/CN			−.26	.35	[−.96, .44]	−.09	.01
		mxMCI/CN			−.64	.33	[−1.29, .01]	−.25	.04
		aMCI/SbCI			.34	.32	[−.30, .98]	.12	.01
		mxMCI/SbCI			−.04	.29	[−.62, .55]	−.01	.00
		mxMCI/aMCI			−.38	.33	[−1.03, .27]	−.15	.01
COM-SR	1		.01	.01					
		Education			.04	.06	[−.07, .15]	.07	.00
		Sex^a^			−.17	.23	[−.62, .29]	−.07	.00
	2		.22***	.21***					
		SbCI/CN			−.85	.35	[−1.55, −.15]	−.29*	.05
		aMCI/CN			−1.19	.40	[−1.98, −.41]	−.35**	.07
		mxMCI/CN			−1.85	.37	[−2.58, −1.12]	−.59***	.20
		aMCI/SbCI			−.35	.36	[−1.06, .37]	−.10	.01
		mxMCI/SbCI			−1.00	.33	[−1.65, −.35]	−.32**	.08
		mxMCI/aMCI			−.66	.37	[−1.38, .07]	−.21	.03
COPY-DE	1		.01	.01					
		Education			.06	.05	[−.02, .01]	.11	.00
		Sex^a^			.11	.20	[−.09, .07]	.06	.00
	2		.12*	.10*					
		SbCI/CN			−.68	.33	[.01, .15]	.−.26*	.06
		aMCI/CN			−.35	.37	[.10, .26]	−.12	.26
		mxMCI/CN			−1.06	.34	[−.18, −.05]	−.39**	.07
		aMCI/SbCI			.33	.34	[−.33, 1.00]	.11	.01
		mxMCI/SbCI			−.38	.31	[−.99, .22]	−.14	.01
		mxMCI/aMCI			−.72	.34	[−1.39, −.04]	−.26*	.04
COPY-IP	1		.02	.02					
		Education			−.03	.04	[−.11, .05]	−.07	.01
		Sex^a^			.15	.16	[−.17, .47]	.09	.01
	2		.11*	.10*					
		SbCI/CN			−.61	.27	[−1.14, −.08]	−.29*	.05
		aMCI/CN			−.28	.30	[−.87, .31]	−.11	.01
		mxMCI/CN			−.82	.28	[−1.38, −.27]	−.37**	.08
		aMCI/SbCI			.27	.27	[−.27, .81]	.11	.01
		mxMCI/SbCI			−.28	.25	[−.77, .21]	.−13	.01
		mxMCI/aMCI			−.55	.28	[−1.10, .00]	−.25	.04
COPY-SCM	1		.01	.01					
		Education			.03	.04	[−.06, .12]	.06	.00
		Sex^a^			−.14	.17	[−.48, .19]	−.09	.01
	2		.07	.06					
		SbCI/CN			−.61	.29	[−1.17, −.04]	−.28*	.04
		aMCI/CN			−.20	.32	[−.84, .44]	−.08	.00
		mxMCI/CN			−.53	.30	[−1.11, .06	−.23	.03
		aMCI/SbCI			.41	.29	[−.17, .99]	.16	.02
		mxMCI/SbCI			.08	.27	[−.45, .61]	.04	.00
		mxMCI/aMCI			−.33	.30	[−.91, .26]	−.14	.01
COPY-SR	1		.00	.00					
		Education			−.03	.05	[−.13, .08]	−.05	.00
		Sex^a^			−.04	.21	[.86, −.44]	−.02	.00
	2		.07	.07					
		SbCI/CN			−.12	.35	[−.81, .56]	−.05	.00
		aMCI/CN			.26	.39	[−.51, 1.04]	.09	.00
		mxMCI/CN			−.65	.36	[−1.4, .06]	−.23	.03
		aMCI/SbCI			.39	.36	[−.32, 1.09]	.13	.01
		mxMCI/SbCI			−.53	.32	[−1.17, .11]	−.19	.03
		mxMCI/aMCI			−.91	.36	[−1.63, −20]	−.33*	.06

*Note.* n = 105; CI = confidence interval; sr^2^ = squared semi-partial correlation coefficient;

a 1 = female, 0 = male; DCTclock = total command/copy score; DE = drawing Efficiency; SCM = Simple/Complex Motor Operations; IP = Information Processing; SP = Spatial Reasoning; CN = normal cognition; SbCI = subtle cognitive impairment; aMCI = amnestic mild cognitive impairment; mx/dysMCI = mixed/dysexecutive cognitive impairment.

* p< .05

** p< .01

*** p< .001.

## References

[R1] Ahmed S, Brennan L, Eppig J, Price CC, Lamar M, DelanoWood L. . . . & Libon DJ (2016). Visuoconstructional impairment in subtypes of mild cognitive impairment. Applied Neuropsychology Adult, 23(1), 43–52. doi: 10.1080/23279095 26397732 PMC5927360

[R2] Binaco R, Calzaretto N, Epifano J, McGuire S, Umer M, Emrani S, . . . & Polikar R. (2020). Machine learning analysis of digital clockdrawing performance for differential classification of mild cognitive impairment subtypes versus Alzheimer’s disease. Journal of the International Neuropsychological Society, 1–11. doi: 10.1017/S1355617720000144 32200771

[R3] Bondi MW, Edmonds EC, Jak AJ, Clark LR, Delano-Wood L, McDonald CR, . . . & Salmon DP (2014). Neuropsychological criteria for mild cognitive impairment improves diagnostic precision, biomarker associations, and progression rates. Journal of Alzheimer’s Disease, 42(1), 275–289. doi: 10.3233/JAD-140276 PMC4133291 24844687

[R4] Cosentino S, Jefferson A, Chute DL, Kaplan E, & Libon DJ (2004). Clock drawing errors in dementia: Neuropsychological and neuroanatomical considerations. Cognitive Behavioral Neurology, 17(2), 74–84. doi: 10.1097/01.wnn.0000119564.08162.46 15453515

[R5] Davis R, Libon DJ, Au R, Pitman D, & Penney DL (2014). THink: Inferring cognitive status from subtle behaviors. In Proc Conf AAAI Artificial. Intelligence, 2014, (pp. 2898–2905). PMC4825804 27066307

[R6] Davoudi A, Dion C, Amini S, Tighe PJ, Price CC, Libon DJ, & Rashidi P. (2021). Classifying non-dementia and alzheimer’s disease/vascular dementia patients using kinematic, time-based, and visuospatial parameters: The digital clock drawing test. Journal of Alzheimer’s Disease, 82(1), 47–57. doi: 10.3233/JAD-201129 PMC8283934 34219737

[R7] Delis DC, Kramer JH, Kaplan E, & Ober BA (2000). The California Verbal Learning Test-II. New York: Psychology Corporation.

[R8] Devlin KN, Brennan L, Saad L, Giovannetti T, Hamilton RH, Wolk DA, Xie SX, & Mechanic-Hamilton D. (2021). Diagnosing mild cognitive impairment among racially diverse older adults: Comparison of consensus, actuarial, and statistical methods. Journal of Alzheimer’s Disease, 85(2), 627–644. doi: 10.3233/JAD-210455 PMC8820242 34864658

[R9] Dion C, Arias F, Amini S, Davis R, Penney D, Libon DJ, & Price CC (2020). Cognitive correlates of digital clock drawing metrics in older adults with and without mild cognitive impairment. Journal of Alzheimer’s Disease, 75(1), 73–83. doi: 10.3233/JAD-191089 PMC7217723 32250300

[R10] Dugger BN, Davis K, Malek-Ahmadi M, Hentz JG, Sandhu S, Beach TG, Adler CH, Caselli RJ, Johnson TA, Serrano GE, Shill HA, Belden C, Driver-Dunckley E, Caviness JN, Sue LI, Jacobson S, Powell J, & Sabbagh MN (2015). Neuropathological comparisons of amnestic and nonamnestic mild cognitive impairment. BMC Neurology, 15, 146. 10.1186/s12883-015-0403-4 26289075 PMC4545878

[R11] Edmonds EC, Delano-Wood L, Galasko DR, Salmon DP, & Bondi MW (2015). Subtle cognitive decline and biomarker staging in preclinical Alzheimer’s disease. Journal of Alzheimer’s Disease, 47(1), 231–242. doi: 10.3233/JAD-150128 PMC4634634 26402771

[R12] Emrani S, Libon DJ, Lamar M, Price CC, Jefferson AL, Gifford KA, . . . & Au R. (2018). Assessing working memory in mild cognitive impairment with serial order recall. Journal of Alzheimer’s Disease, 61(3), 917–928. doi: 10.3233/JAD-170555 PMC5901759 29254087

[R13] Emrani S, Lamar M, Price C, Baliga S, Wasserman V, Matusz E, . . . & Libon DJ (2021a). Neurocognitive constructs underlying executive control in statistically-determined Mild Cognitive Impairment. Journal of Alzheimer’s Disease, 82(1), 5–16. doi: 10.3233/JAD-201125 34219736

[R14] Emrani S, Lamar M, Price CC, Baliga S, Wasserman V, Matusz E, . . . & Libon D. (2021b). Assessing the capacity for mental manipulation in patients with statistically-determined mild cognitive impairment using digital technology. Exploration of Medicine, 2, 86–97. doi: 10.37349/emed.2021.00034

[R15] Eppig J, Wambach D, Nieves C, Price CC, Lamar M, Delano-Wood L, Giovannetti T, Bettcher BM, Penney DL, Swenson R, Lippa C, Kabasakalian A, Bondi MW, & Libon DJ (2012). Dysexecutive functioning in mild cognitive impairment: derailment in temporal gradients. Journal of the International Neuropsychological Society, 18(1), 20–28. 22014116 10.1017/S1355617711001238 PMC3315354

[R16] Ferman TJ, Smith GE, Kantarci K, Boeve BF, Pankratz VS, Dickson DW, Graff-Radford NR, Wszolek Z, Van Gerpen J, Uitti R, Pedraza O, Murray ME, Aakre J, Parisi J, Knopman DS, & Petersen RC (2013). Nonamnestic mild cognitive impairment progresses to dementia with Lewy bodies. Neurology, 81(23), 2032–2038. doi: 10.1212/01.wnl.0000436942.55281.47 24212390 PMC3854825

[R17] Grundman M, Petersen RC, Ferris SH, Thomas RG, Aisen PS, Bennett DA, Foster NL, Jack CR Jr, Galasko DR, Doody R, Kaye J, Sano M, Mohs R, Gauthier S, Kim HT, Jin S, Schultz AN, Schafer K, Mulnard R, van Dyck CH, . . . Alzheimer’s Disease Cooperative Study (2004). Mild cognitive impairment can be distinguished from Alzheimer disease and normal aging for clinical trials. Archives of Neurology, 61(1), 59–66. doi: 10.1001/archneur.61.1.59 14732621

[R18] Guillozet AL, Weintraub S, Mash DC, & Mesulam MM (2003). Neurofibrillary tangles, amyloid, and memory in aging and mild cognitive impairment. Archives of Neurology, 60(5), 729–736. doi: 10.1001/archneur.60.5.729 12756137

[R19] Hazan E, Frankenburg F, Brenkel M, & Shulman K. (2018). The test of time: A history of clock drawing. International Journal of Geriatric Psychiatry, 33(1), e22–e30. doi: 10.1002/gps.4731 28556262

[R20] Heaton RK, Miller S, Taylor M, Grant I (2004) Revised comprehensive norms for an expanded Halstead-Reitan Battery: Demographically adjusted neuropsychological norms for African American and Caucasian adults scoring programs. Psychological Assessment Resources.

[R21] Jak A, Bondi MW, Delano-Wood L, Wierenga C, Corey-Bloom J, Salmon DP, Delis DC (2009). Quantification of five neuropsychological approaches to defining mild cognitive impairment. The American Journal of Geriatric Psychiatry, 17, 456–465. doi: 10.1097/JGP.0b013e31819431d5 PMC2743175 19390294

[R22] Kaplan E, Goodglass H, & Weintraub S. (1983). The Boston Naming Test. Philadelphia: Lea and Febiger.

[R23] Kozora E. & Collum CM (1994). Qualitative features of clock drawings in normal aging and Alzheimer’s disease. Assessment, 1(2), 179–187. doi: 10.1177/1073191194001002008 9465148

[R24] Lamar M, Price CC, Davis KL, Kaplan E, & Libon DJ (2002). Capacity to maintain mental set in dementia. Neuropsychologia, 40(4), 435–445. 11684176 10.1016/s0028-3932(01)00125-7

[R25] Lamar M, Ajilore O, Leow A, Charlton R, Cohen J, GadElkarim J, . . . & Kumar A. (2016). Cognitive and connectome properties detectable through individual differences in graphomotor organization. Neuropsychologia, 85, 301–309. 27037044 10.1016/j.neuropsychologia.2016.03.034 PMC4853274

[R26] Lee DY, Seo EH, Choo IH, Kim SG, Lee JS, Lee DS, . . . & Woo JI (2008). Neural correlates of the Clock Drawing Test performance in Alzheimer’s disease: A FDGPET study. Dementia and Geriatric Cognitive Disorders, 26(4), 306–313. 18841015 10.1159/000161055

[R27] Libon DJ, Bondi MW, Price CC, Lamar M, Eppig J, Wambach DM, . . . & Penney DL (2011). Verbal serial list learning in mild cognitive impairment: A profile analysis of interference, forgetting, and errors. Journal of the International Neuropsychological Society : JINS, 17(5), 905–914. 21880171 10.1017/S1355617711000944 PMC3315271

[R28] Libon DJ, Malamut BL, Swenson R, Sands LP, & Cloud BS (1996). Further analyses of clock drawings among demented and nondemented older subjects. Archives of Clinical Neuropsychology, 11(3), 193–205. 14588923

[R29] Libon DJ, Penney DL, Davis R, Tabby DS, Eppig J, Nieves C, . . . & Garret KD (2014). Deficits in processing speed and decision making in relapsing-remitting multiple sclerosis: The digit clock drawing test (dCDT). Journal of Multiple Sclerosis, 1(2), 113. doi: 10.4172/2376-0389.1000113

[R30] Müller S, Herde L, Preische O, Zeller A, Heymann P, Robens S, Elbing U, & Laske C. (2019). Diagnostic value of digital clock drawing test in comparison with CERAD neuropsychological battery total score for discrimination of patients in the early course of Alzheimer’s disease from healthy individuals. Scientific Reports, 9(1), 3543. 10.1038/s41598-019-40010-0 30837580 PMC6400894

[R31] Piers RJ, Devlin KN, Ning B, Yulin L, Wasserman B, Massaro JM, . . . & Libon DJ (2017). Age and graphomotor decision making assessed with the digital clock drawing test: The Framingham Heart Study. Journal of Alzheimer’s Disease, 60(4), 1611–1620. doi: 10.3233/JAD-170444. PMC7286350 29036819

[R32] Price CC, Cunningham H, Coronado N, Freedland A, Cosentino S, Penney DL, Penisi A, Bowers D, Okun MS, & Libon DJ (2011). Clock drawing in the Montreal Cognitive Assessment: Recommendations for dementia assessment. Dementia and Geriatric Cognitive Disorders, 31(3), 179–187. 21389719 10.1159/000324639 PMC3065510

[R33] Reitan RM & Wolfson D. (1985). The Halstead-Reitan neuropsychological test battery: Theory and clinical interpretation, (4th ed.). Tucson, AZ: Reitan Neuropsychology.

[R34] Rentz DM, Papp KV, Mayblyum DV, Sanchez JS, Klein H, Souillard-Mandar W, . . . & Johnson KA (2021). Association of digital clock drawing with PET amyloid and tau pathology in normal older adults. Neurology, 96(14), e1844–e1854. 33589537 10.1212/WNL.0000000000011697 PMC8105970

[R35] Royall DR, Cordes JA, & Polk M. (1998). CLOX: An executive clock drawing task. Journal of Neurology, Neurosurgery, and Psychiatry, 64(5), 588–594. doi: 10.1136/jnnp.64.5.588 9598672 PMC2170069

[R36] Schejter-Margalit T, Kizony R, Shirvan J, Cedarbaum JM, Bregman N, Thaler A, Giladi N, & Mirelman A. (2021). Quantitative digital clock drawing test as a sensitive tool to detect subtle cognitive impairments in early-stage Parkinson’s disease. Parkinsonism & Related Disorders, 90, 84–89. doi: 10.1016/j.parkreldis.2021.08.002 34416663

[R37] Schneider JA, Arvanitakis Z, Leurgans SE, & Bennett DA (2009). The neuropathology of probable Alzheimer disease and mild cognitive impairment. Annals of Neurology, 66(2), 200–208. 10.1002/ana.21706 19743450 PMC2812870

[R38] Shoyama M, Nishioka T, Okumura M, Kose A, Tsuji T, Ukai S, & Shinosaki K. (2011). Brain activity during the clock-drawing test: Multichannel near-infrared spectroscopy study. Applied Neuropsychology, 18(4), 243–251. doi: 10.1080/09084282.2011.595450 22074062

[R39] Souillard-Mandar W, Davis R, Rudin C, Au R, Libon DJ, Swenson R, (2016). Learning classification models of cognitive conditions from subtle behaviors in the digital clock drawing test. Machine Learning, 102(3): 393–441. 27057085 10.1007/s10994-015-5529-5 PMC4821477

[R40] Spreen O. & Strauss E. (1990). Compendium of neuropsychological tests. New York, NY: Oxford University Press.

[R41] Tabachnick BG & Fidell LS (2013). Using multivariate statistics (6th ed.). Boston, MA: Pearson.

[R42] Thomann PA, Toro P, Santos VD, Essig M, & Schröder J, 2008. Clock drawing performance and brain morphology in mild cognitive impairment and Alzheimer’s disease. Brain and Cognition, 67, 88–93. doi: 10.1016/j.bandc.2007.11.008 18215449

[R43] Wechsler D. (1997).WAIS-III: Administration and scoring manual: Wechsler adult intelligence scale. San Antonio, TX: Psychological Corporation.

[R44] Wiggins ME, Dion C, Formanski EF, Davoudi AD, Amini S, Heilman KM, Penney D, Davis R, Garvan CW, Arnaoutakis GJ, Tighe P, Libon DJ, & Price CC (2021). Proof of concept: Digital clock drawing behaviors prior to transcatheter aortic valve replacement may predict length of hospital stay and cost of care. Exploration of Medicine, 2, 110–121. 34263257 10.37349/emed.2021.00036 PMC8276939

