# Supplementary material for: Dissociating Statistically Determined Normal Cognitive Abilities and Mild Cognitive Impairment Subtypes with DCTclock
Source: J Int Neuropsychol Soc. Author manuscript; Available in PMC 2024 Jun 24. (PMC11194727; doi:10.1017/S1355617722000091)
Supplement: supplemental tables [file NIHMS1968180-supplement-supplemental_tables.docx]

Supplemental Table 1.

| **DCTclock^TM^ Composite subscale variables** | **Description** | **Member composite group** |
| --- | --- | --- |
| Stroke Count Conformity | The deviation from the expected number of pen strokes in the drawing, normed with respect to cognitively healthy individuals. | Drawing Efficiency composite |
| Total Time | The total time spent completing the drawing, normed with respect to cognitively healthy individuals | Drawing Efficiency composite |
| Ink Length | The total length in millimeters of the ink used in the drawing, normed with respect to cognitively healthy individuals | Drawing Efficiency composite |
| Drawing Size | The size in millimeters of the clock face circle, normed with respect to cognitively healthy individuals | Drawing Efficiency composite |
| Drawing Process Efficiency | A measure that combines Ink Length and Total Time, normed with respect to cognitively healthy individuals | Drawing Efficiency composite |
| Noise | A measure of the drawing that includes overwriting or non-standard pen strokes, normed with respect to cognitively healthy individuals | Drawing Efficiency composite |
| Percent Ink Time | The percentage of the test time spent actively drawing, normed with respect to cognitively healthy individuals | Simple Motor composite |
| Average Speed | The average speed of the pen during the drawing of the clock face, normed with respect to cognitively healthy individuals | Simple Motor composite |
| Max Speed | The maximum speed of the pen during the drawing of the clock face, normed with respect to cognitively healthy individuals | Simple Motor composite |
| Initiation Speed | The speed of the pen when beginning to draw the clock face, normed with respect to cognitively healthy individuals | Simple Motor composite |
| Termination Speed | The speed of the pen when finishing the clock face, normed with respect to cognitively healthy individuals | Simple Motor composite |
| Oscillatory Motion | A measure of how much the motion of the pen deviates from a smooth motion during the drawing process, normed with respect to cognitively healthy individuals | Simple Motor composite |
| Percent Think Time | The percentage of the test time spent "thinking" (i.e., holding the pen but not actively drawing), normed with respect to cognitively healthy individuals | Information Processing composite |
| Average Latency | The average duration of the delays between each pen stroke, normed with respect to cognitively healthy individuals | Information Processing composite |
| Latency Variability | The variability in the delays throughout the drawing process on the copy clock, normed with respect to cognitively healthy individuals | Information Processing composite |
| Relative Long Latency | The combined duration of delays that are long compared to the participant’s average individual performance on the copy clock, normed with respect to cognitively healthy individuals | Information Processing composite |
| Long Latency Count | The total number of delays in the drawing that are longer than average on the copy clock, normed with respect to cognitively healthy individuals | Information Processing composite |
| Longest Latency | The duration of the longest delay in drawing the copy clock, normed with respect to cognitively healthy individuals | Information Processing composite |
| Clockface Circularity | A measure of the roundness of the clock face circle on the copy clock, normed with respect to cognitively healthy individuals | Spatial Reasoning composite |
| Component Placement | A measure of the spatial relation of the drawing components on the copy clock, normed with respect to cognitively healthy individuals | Spatial Reasoning composite |
| Vertical Spatial Placement | A measure of the vertical position on the page of the drawing of the copy clock, normed with respect to cognitively healthy individuals | Spatial Reasoning composite |
| Horizontal Spatial Placement | A measure of the horizontal position on the page of the drawing on the copy clock, normed with respect to cognitively healthy individuals | Spatial Reasoning composite |
